# Neurobehavioral Protection by Prebiotic Formulations in a Scopolamine-Induced Cognitive Impaired Zebrafish Model

**DOI:** 10.3390/life15081268

**Published:** 2025-08-11

**Authors:** Emanuel Vamanu, Ionela Avram, Diana Roxana Pelinescu, Hesham R. El-Seedi, Lucian Hritcu, Ion Brinza, Laura-Dorina Dinu, Razvan Stefan Boiangiu

**Affiliations:** 1 Faculty of Biotechnology, University of Agronomic Sciences and Veterinary Medicine of Bucharest, 011464 Bucharest, Romania; 2 Anoom Laboratories SRL, Pipera-Tunari Road 1 (Euromaster Building), 077190 Voluntari, Romania; 3 Department of Genetics, University of Bucharest, 050107 Bucharest, Romania; 4 Department of Chemistry, Faculty of Science, Islamic University of Madinah, Madinah 42351, Saudi Arabia; halsaeedi@iu.edu.sa; 5 Department of Biology, Faculty of Biology, Alexandru Ioan Cuza University of Iasi, 700506 Iasi, Romania; razvan.boiangiu@uaic.ro; 6 Faculty of Sciences, Lucian Blaga University of Sibiu, 550024 Sibiu, Romania; ion.brinza@ulbsibiu.ro

**Keywords:** prebiotics, zebrafish, memory, gut–brain axis, neuroinflammation, scopolamine

## Abstract

The present work evaluates the influence of two prebiotic formulations—P1 (ColonX) and P4 (a product containing AnXietate extract), at concentrations of 3 and 6 mg/L—on scopolamine (SCOP, 100 μM)-induced cognitive dysfunction and anxiety-related behaviors in adult zebrafish (Danio rerio). To assess behavioral alterations, wild-type fish were subjected to the novel tank assay (NTT), Y-maze study, and novel object recognition test protocol (NOR). The formulations were examined for potential anti-inflammatory activity and cytotoxicity. In parallel, in vitro assays were performed to evaluate cytotoxic and anti-inflammatory effects. The results indicate that both prebiotic formulations effectively mitigated SCOP-induced behavioral impairments and improved cognitive performance in zebrafish. Furthermore, the prebiotic formulation P4 showed significant anti-inflammatory activity without inducing cytotoxicity. The study was conducted following ethical guidelines, ensuring scientific rigor and integrity. These findings highlight the therapeutic potential of prebiotics in alleviating anxiety and cognitive deficits, with promising implications for the management of neuropsychiatric disorders.

## 1. Introduction

In recent years, the zebrafish (*Danio rerio*) has emerged as a prominent in vivo model in neuroscience and neuropharmacological research, offering a powerful platform for screening bioactive compounds with anxiolytic and cognitive-enhancing potential [1,2]. Its genetic and neuroanatomical homology with mammals, including conservation of neurotransmitter systems, combined with its small size, optical transparency, cost-effectiveness, and suitability for high-throughput screening, make it an ideal organism for modeling human neurological disorders [3,4].

Zebrafish have proven especially useful for investigating the gut–brain axis—a two-way communication network that connects the gastrointestinal tract and the central nervous system via neural, hormonal, and immune signaling pathways [5]. Recent evidence confirms that alterations in gut microbiota can significantly impact brain function, behavior, and neuroinflammation [6]. Notably, administration of specific microbial strains such as *Paraburkholderia sabiae* has been shown to reduce anxiety-like behavior in zebrafish by modulating microbial composition and increasing brain taurine levels, thereby reinforcing the utility of this model in microbiota–brain studies [7].

Anxiety and memory impairments are growing public health concerns, with rising global prevalence and significant social and economic burdens [8]. Pharmacological treatments often show limited efficacy, are associated with side effects such as sedation and dependence, and fail to address underlying pathophysiological mechanisms, including neuroinflammation and oxidative stress [9,10]. This has driven interest in discovering safer, multi-targeted natural products that may act through both central and peripheral mechanisms, including modulation of the gut–brain axis [11,12].

Scopolamine (SCOP), an antagonist of muscarinic cholinergic receptors, is commonly employed in both zebrafish and rodent models to mimic cognitive impairment and anxiety-like symptoms, making it a well-established model for testing neuroprotective compounds [13]. Although multiple synthetic drugs have been assessed in this model, the effects of microbiota-modulating or plant-derived compounds remain underexplored [14,15]. Recent studies have shown that essential oils and plant extracts, such as those from *Solanum macrocarpon* and *Origanum majorana*, can ameliorate SCOP-induced behavioral and oxidative changes, highlighting the potential of phytochemicals in neuroprotection [16,17].

Over the last few years, growing evidence has highlighted the important role of the gut–brain axis in neuropsychiatric disorders [12]. The gut microbiota complex ecosystem of microorganisms from the gastrointestinal tract directly influences brain function and behavior [18]. This pathway opens novel therapeutic ways to control brain health. The impact of possible compounds on the gut microbiota is essential for a modulating process and a clear understanding of their neuropharmacological effects [6].

The current study aimed to evaluate the neuroprotective potential of two commercial natural formulations, P1 (ColonX) and P4 (AnXietate), both developed by Anoom Laboratories SRL, Romania, in counteracting SCOP-induced anxiety-like behavior and cognitive deficits in adult zebrafish. A secondary objective was to assess their in vitro anti-inflammatory effects, providing insights into their potential to modulate the gut–brain axis. By addressing behavioral, biochemical, and immunological endpoints, this study contributes to the growing body of literature on microbiota-influencing compounds and their role in managing neuropsychiatric conditions.

## 2. Materials and Methods

### 2.1. Plant-Based Materials

The tested formulations were obtained from SC Anoom Biolaboratories SRL (Voluntari, Romania). The P1 sample corresponds to the ColonX product, while P4 represents the base extract used in the commercial product AnXietate (available at www.greenbiom.ro). Both products were prepared using a previously established extraction protocol, as described by Avram et al. [19].

### 2.2. Zebrafish Husbandry and Experimental Design

Adult wild-type zebrafish (*Danio rerio*, Tubingen strain), aged 6–8 months and exhibiting the short-fin phenotype (equal male-to-female ratio), were sourced from the European Zebrafish Resource Center at the Institute of Toxicology and Genetics, Germany. The animals were housed in the zebrafish facility of the Faculty of Biology, Alexandru Ioan Cuza University of Iași. Groups of 10 fish were kept in 10 L tanks under standardized conditions: 27 ± 1 °C water temperature, pH 7.0 ± 0.3, dissolved oxygen at 6.5 ± 0.5 mg/L, and a 14:10 h light/dark cycle, in accordance with previously established protocols [20]. The fish were fed ad libitum using an automated feeder with Norwin Norvital flakes (Norwin, Gadstrup, Denmark) three times daily at 07:00, 14:00, and 19:00. All food was consumed within 10 min of distribution.

Prior to experiments, fish were acclimated for two weeks and maintained under quarantine and monitored for health status. They were then randomly assigned into the following experimental groups (*n* = 10 per group): control, SCOP (100 µM), and P1 and P4 treatment at 3 mg/L and 6 mg/L. Treatments consisted of exposure to SCOP (100 µM), either alone or in combination with prebiotic formulations P1 or P4 at concentrations of 3 mg/L or 6 mg/L, administered via immersion for seven consecutive days. Control groups received standard tank water. Additionally, sample size justification (*n* = 10 zebrafish per group) was confirmed using InVivoStat and an R-based statistical analysis package [21]. Using a significance threshold of 0.05, the statistical power to detect a biologically meaningful difference of 20% was calculated to be 95%. Data analysis, reporting, and experimental design were conducted in accordance with the PREPARE and ARRIVE guidelines [22] for planning and organizing animal testing and research, respectively. All statistical analyses were conducted blindly, ensuring that outcomes were not influenced by the experimenters. The study protocol was reviewed and approved by the Ethics Committee for Animal Research at the Faculty of Biology, Alexandru Ioan Cuza University of Iași, Romania (protocol code BIO-UAIC-2023-1714; approval date: 6 July 2023). All procedures complied with Directive 2010/63/EU [23] of the European Parliament and Council on the protection of animals used for scientific purposes. Animal welfare was prioritized throughout the study, and efforts were made to minimize the number of animals used.

### 2.3. Behavioral Testing Procedure

#### 2.3.1. Novel Tank Diving Test

The novel tank diving test (NTT), originally validated by Cachat et al. [24], is a widely accepted method for evaluating anxiety-related behavior and locomotion in zebrafish. In the present study, fish were tested in a 1.5 L trapezoidal tank (15.2 × 27.9 × 7.1 cm), which was virtually segmented into upper and lower zones using a horizontal divider. Individual fish were placed into the tank and allowed to explore for 6 min. Behavioral parameters were recorded and analyzed using ANY-maze^®^ software v. 7.48 (Stoelting Co., Wood Dale, IL, USA).

Locomotor activity was assessed using several parameters, including time spent in the top zone (s), distance traveled within the top zone (m), total distance traveled (m), and velocity (m/s). Anxiety-like behavior was assessed using standard parameters in the NTT, including the number of entries into the top zone, the time spent in the top zone (s), and latency to first entry into the top zone (s). These metrics are validated indicators of anxiety-related responses in zebrafish.

#### 2.3.2. Y-Maze Test

The Y-maze test was employed to assess novelty recognition, short-term spatial memory, and locomotor behavior in zebrafish, following established protocols [25]. Behavioral testing was performed in a 3-L Y-shaped glass tank consisting of three identical arms (25 × 8 × 15 cm). The arms were designated as follows: a start arm (consistently open), a novel arm (initially closed during the training phase), and a third arm that remained open throughout the experiment. Each fish was individually placed in the start arm for a 5 min training session, during which the novel arm remained closed. After a 1 h inter-trial interval, a second 5 min session was conducted with all arms accessible, including the previously closed novel arm.

Behavioral data were recorded and analyzed using ANY-maze^®^ software v. 7.48 (Stoelting Co., Wood Dale, IL, USA). Novelty-seeking behavior was assessed by calculating the percentage of time spent in the novel arm relative to total arm time, while the spontaneous alternation percentage served as an indicator of short-term memory. Spontaneous alternation refers to the natural tendency of zebrafish to explore a new arm of the maze rather than returning to one that was recently visited. It is usually expressed as percentage, calculated using the following formula: [number of alternations/total arm entries − 2] × 100. Locomotor activity was evaluated through several parameters, including the number of arm entries, total distance traveled (m), turn angle (°), and number of line crossings. Turn angle refers to the angular change in direction between consecutive movements of the zebrafish as it swims through the Y-maze. Line crossing refers to the number of times the zebrafish crosses predefined virtual lines or zones in the Y-maze arms or center.

#### 2.3.3. Novel Object Recognition Test

The novel object recognition (NOR) test was employed to assess recognition memory in zebrafish, as previously described [26]. During the habituation phase, fish were placed individually into a 30 × 30 × 30 cm glass tank (20 L) filled with 6 cm of water from the home tank without objects for 5 min twice a day (5 h between habituation sessions) over three consecutive days to acclimate them to the environment.

On the fourth day (training phase), the zebrafish were presented with two identical objects (red cubes) for a 10 min exploration period. After a one-hour interval, during the testing phase, one familiar object (FO, red cube) was substituted with a novel object (NO, green cube), and the fish were allowed to explore both for an additional 10 min. Recognition memory was evaluated by calculating the preference for the novel object index using the following formula: [Time exploring NO/(Time exploring FO + Time exploring NO)] × 100. Behavioral activity was tracked and analyzed using ANY-maze^®^ software v. 7.48 (Stoelting Co., Wood Dale, IL, USA).

### 2.4. Assessment of Cytotoxicity and Anti-Inflammatory Effects

This study aimed to investigate the cytotoxic and immunomodulatory effects of prebiotic extracts. Human colorectal adenocarcinoma HT-29 cells were treated with the extracts at concentrations of 10% and 2% for 24 h under standard culture conditions. Cell viability was assessed using the 3-(4,5-dimethylthiazol-2-yl)-2,5-diphenyltetrazolium bromide (MTT) assay [27]. Following treatment, MTT reagent was added and incubated for 4 h to allow for the reduction of MTT to formazan crystals by metabolically active cells. The resulting formazan was dissolved using dimethyl sulfoxide (DMSO) [28], and absorbance was measured at 540 nm using a microplate reader.

To evaluate immunomodulatory activity, peripheral blood mononuclear cells (PBMCs) were isolated from healthy donors using density gradient centrifugation [29]. The cells were treated with the same extract concentrations and stimulated with lipopolysaccharides (LPSs) to induce an inflammatory response [30]. After incubation, culture supernatants were collected, and cytokine levels, including interleukin (IL)-1β, IL-6, IL-10, and tumor necrosis factor alpha (TNF-α), were quantified using enzyme-linked immunosorbent assay (ELISA) kits [31]. This combined in vitro approach provides insight into the cytotoxic potential and immune-modulating properties of the tested prebiotic extracts.

### 2.5. Statistical Analyses

All results are expressed as the mean ± standard error of the mean (SEM). Data were analyzed using GraphPad Prism version 9.0 (GraphPad Software, San Diego, CA, USA). The normality of the data distribution was assessed using the Shapiro–Wilk test. For normally distributed data, one-way analysis of variance (ANOVA) followed by Tukey’s HSD post hoc test was performed to compare multiple groups. For non-normally distributed data, appropriate nonparametric tests were applied. Statistical significance was defined as *p* < 0.05.

## 3. Results

### 3.1. P1 and P4 Reduced Anxiety-Like Responses Induced by Scopolamine in the Novel Diving Test

**Figure 1:**
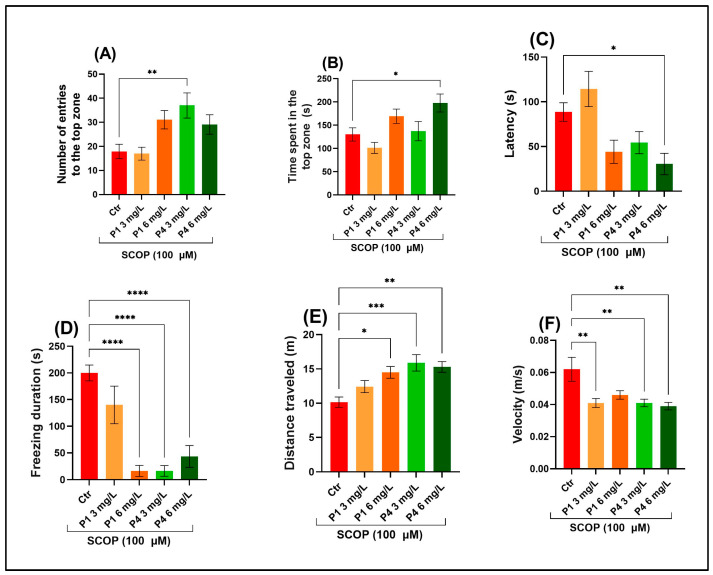
Behavioral effects of P1 and P4 at concentrations of 3 and 6 mg/L on zebrafish exposed to scopolamine (SCOP, 100 µM) in the novel tank test (NTT). Parameters assessed included (**A**) frequency of entries into the top zone, (**B**) total duration spent in the top zone (seconds), (**C**) latency to first entry (seconds), (**D**) freezing time (seconds), (**E**) total distance moved (meters), and (**F**) average swimming speed (meters per second). Data are presented as mean values ± S.E.M. with *n* = 10 animals per group. Statistical analysis was performed using the Tukey HSD post hoc test: * *p* < 0.05, ** *p* < 0.001, *** *p* < 0.0001, and **** *p* < 0.00001.

The novel tank diving test (NTT) was used to determine whether treatment with P1 and P4 could mitigate the anxiety-like effects triggered by SCOP administration. One-way ANOVA showed a significant main effect of treatment on the number of entries into the top zone of the tank, F (4, 45) = 5.07, *p* < 0.001) (Figure 1A), reflecting treatment-related modulation of exploratory behavior. There was also a significant main effect of treatment on the time spent in the top zone of the tank, F (4, 45) = 4.99, *p* < 0.001 (Figure 1B), suggesting altered anxiety-like behavior across treatment groups. In addition, treatment significantly affected the latency to enter the top zone, F (4, 45) = 6.13, *p* < 0.0001 (Figure 1C), demonstrating treatment-related changes in behavioral initiation or anxiety response. The ANOVA presented a substantial main effect after in vivo treatment on the freezing duration, F (4, 45) = 4.30, *p* < 0.001 (Figure 1D). This outcome implies that the treatments significantly influenced freezing behavior, a proxy for anxiety. A one-way ANOVA revealed a significant main effect of treatment on the distance travelled, F (4, 45) = 6.69, *p* < 0.001 (Figure 1E), indicating that locomotor activity differed significantly among groups. The treatment exerted another significant main impact, observed for velocity, F (4, 45) = 5.490, *p* < 0.001 (Figure 1F), indicating differences in general locomotor activity among groups. It was evident that in SCOP-administered zebrafish, P1 and P4 significantly decreased the freezing duration at all doses (P1 6 mg/L, P4 3 and 6 mg/L, *p* < 0.00001) (Figure 1D) and decreased the fish’s swim speed across all applied dosages (P1 3 mg/L, *p* < 0.001; P4 3 and 6 mg/L, *p* < 0.001) (Figure 1F), relative to controls. Moreover, P1 and P4 treatment promoted top-zone exploration by shortening the time required to reach it (P4 6 mg/L, *p* < 0.01) (Figure 1C) and amplifying the time dedicated (P4 6 mg/L, *p* < 0.01) (Figure 1B) and the distance traveled (P1 3 mg/L, *p* < 0.01; P4 3 mg/L, *p* < 0.01 and P4 6 mg/L, *p* < 0.01) (Figure 1F).

### 3.2. P1 and P4 Reversed Scopolamine-Induced Memory Deficits in Y-Maze and Novel Object Recognition Tests

**Figure 2:**
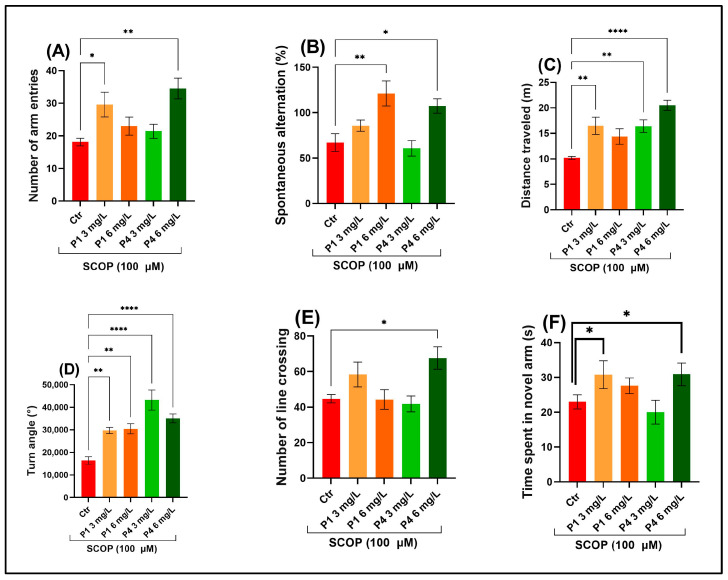
Assessment of the behavioral impact of P1 and P4 at concentrations of 3 and 6 mg/L on zebrafish subjected to scopolamine (SCOP, 100 µM) in the Y-maze test. The following parameters were evaluated: (**A**) total number of arm entries, (**B**) percentage of spontaneous alternations, (**C**) overall distance covered (meters), (**D**) angular turning behavior (degrees), (**E**) frequency of line crossings, and (**F**) duration spent in the novel arm (seconds). Data are presented as mean values ± S.E.M. with *n* = 10 animals per group. Statistical analysis was performed using the Tukey HSD post hoc test: * *p* < 0.05, ** *p* < 0.001, and **** *p* < 0.00001.

The Y-maze test assessed spatial memory and exploratory behavior by measuring zebrafish responses to a novel arm. A significant main effect of treatment was observed for the number of arm entries, F (4, 45) = 5.77, *p* < 0.0001 (Figure 2A), suggesting treatment-induced changes in spatial exploration or working memory-related behavior. Treatment significantly affected intrinsic alternation patterns in behavior, F (4, 45) = 7.25, *p* < 0.0001 (Figure 2B), indicating altered working memory performance under different treatment conditions. There was a significant main effect of treatment on total distance travelled, F (4, 45) = 9.05, *p* < 0.0001 (Figure 2C), indicating that locomotor activity significantly varied across treatment groups. A highly significant main effect of treatment was found for turn angle, F (4, 45) = 14.25, *p* < 0.0001 (Figure 2D), indicating substantial changes in movement trajectory or re-orientation behavior between groups. A one-way ANOVA revealed a significant main effect of treatment on the number of line crossings, F (4, 45) = 4.35, *p* < 0.001 (Figure 2E), further supporting changes in locomotor or exploratory behavior across groups. There was no statistically significant main effect of treatment on the time spent in the novel arm, F (4, 45) = 2.47, *p* < 0.01 (Figure 2F). The Tukey HSD post hoc analysis demonstrated that both P1 and P4 effectively counteracted SCOP-induced deficits in the Y-maze task. Treatments at 3 and 6 mg/L led to a significant rise in arm entries (*p* < 0.01 and *p* < 0.001, respectively; Figure 2A), alternation efficiency score (*p* < 0.001 for P1 at 6 mg/L; *p* < 0.01 for P4 at 6 mg/L; Figure 2B), total distance traveled (*p* < 0.001 for both P1 and P4 at 3 mg/L; *p* < 0.00001 for P4 at 6 mg/L; Figure 2C), and turn angle (*p* < 0.001 for P1 at both doses; *p* < 0.00001 for P4 at both doses; Figure 2D). Additionally, the 6 mg/L concentration of P4 promoted a rise in the total line crossings recorded (*p* < 0.01; Figure 2E), while both P1 at 3 mg/L and P4 at 6 mg/L significantly extended the cumulative time engaged in the novel arm (*p* < 0.01; Figure 2F). Collectively, these results indicate that P1 and P4 alleviate SCOP-induced disruptions in spatial working memory and promote exploratory behavior, highlighting their potential cognitive benefits.

**Figure 3:**
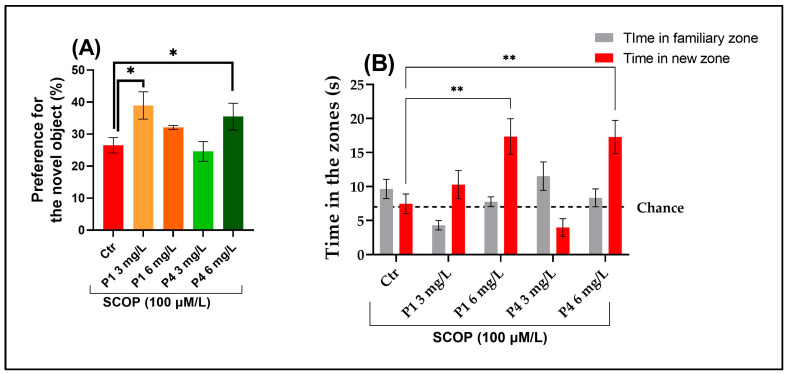
Effect of treatment on (**A**) preference for the novel object behavior and (**B**) exploration time in the familiar versus novel object zones in the NOR test. Results are presented as mean ± SEM, with a sample size of 10 individuals per group. Statistical comparisons were performed using Tukey’s HSD post hoc analysis, where * indicates *p* < 0.01 and ** indicates *p* < 0.001.

Zebrafish recognition memory was evaluated based on their interaction duration with the NO assay compared to a familiar one (FO). One-way ANOVA presented a highly significant treatment effect on object preference behavior (F (4, 45) = 15.59, *p* < 0.0001; Figure 3A), indicating notable differences in preference scores among experimental groups. Additionally, a significant interaction between treatment group and object zone was observed (F (4, 90) = 9.42, *p* < 0.0001; Figure 3B), suggesting that the treatment differentially affected exploration time depending on the object type. Overall, zebrafish spent significantly more time in the novel object zone compared to the familiar one, supporting a robust preference for novelty. Post hoc analysis using Tukey’s HSD test demonstrated that treatment with P4 (6 mg/L) significantly enhanced recognition memory, as evidenced by increased preference for the novel object (*p* < 0.01; Figure 3A). Furthermore, P1 (6 mg/L) and P4 (6 mg/L) treatment significantly elevated the time spent exploring the novel object (*p* < 0.001; Figure 3B), indicating improved cognitive performance. Although zebrafish treated with P3 (6 mg/L) showed a trend toward increased preference for novel object exploration, the effect did not reach statistical significance compared to the control group, suggesting a more modest or inconsistent impact of this formulation on recognition memory.

### 3.3. Prebiotic Treatment on Cytokine Responses: Evaluation of Cytotoxicity and Anti-Inflammatory Properties of Novel Prebiotic Formulations (P4)

**Figure 4:**
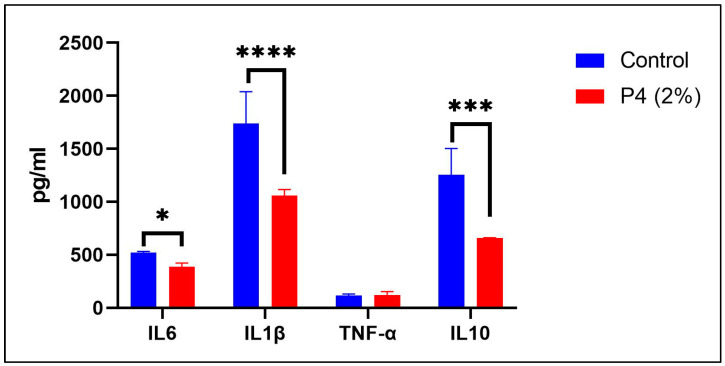
Impact of P4 (2%) prebiotic treatment on cytokine expression in human PBMCs. Results are presented as mean ± S.E.M., with three technical replicates per group (*n* = 3). Group differences were analyzed using one-way ANOVA, followed by Tukey’s HSD post hoc test for multiple comparisons. Statistical significance is denoted as follows: * *p* < 0.01, *** *p* < 0.0001, and **** *p* < 0.00001.

The role of modulating prebiotic treatment (P4) on inflammatory cytokines in human peripheral blood mononuclear cells (PBMCs) was also investigated, the results of which are shown in Figure 4. Treatment-induced changes in inflammatory cytokines were found to be significant based on one-way ANOVA results (F (3, 16) = 8.79, *p* < 0.001; Figure 4), indicating that P4 notably modulated the inflammatory profile among experimental groups. A significant treatment × group interaction was also observed (F (3, 16) = 7.15, *p* < 0.001), suggesting that P4’s anti-inflammatory action was particularly evident under LPS-induced neurotoxic conditions. Further analysis using Tukey’s HSD post hoc test revealed that P4 administration markedly decreased IL-6 (*p* < 0.01), IL-1β (*p* < 0.00001), and IL-10 (*p* < 0.0001) levels when compared to untreated controls (Figure 4). These results suggest that P4 reverses LPS-induced inflammation, which may underline its memory-enhancing effects in the zebrafish model.

**Figure 5:**
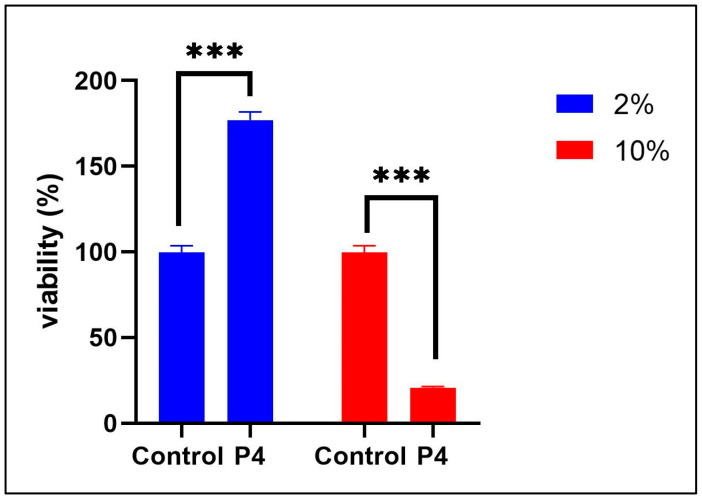
Effect of prebiotic treatment (P4) on HT-29 cell viability assessed by MTT assay. Data are presented mean ± S.E.M., with *n* = 3 technical replicates per group. Statistical analysis was performed using ordinary one-way ANOVA followed by Tukey’s HSD post hoc test. Statistical significance is indicated as *** *p* < 0.0001.

The effect of the prebiotic treatment (P4) on cell viability was further investigated, the results of which are shown in Figure 5. One-way ANOVA showed a significant effect of treatment on cell viability (F (1, 8) = 45.38, *p* < 0.0001; Figure 5), indicating that P4 modulated viability across experimental conditions. A significant treatment × group interaction was also observed (F (1, 8) = 71.65, *p* < 0.0001), suggesting that P4 had both stimulatory and inhibitory effects depending on concentration. Specifically, the Tukey test revealed that P4 at 2% enhanced cell viability (*p* < 0.0001), whereas the 10% concentration led to a marked reduction (*p* < 0.0001). These results suggest that P4 (2%) treatment could be safe and effective for long-term use, reducing inflammation without significant side effects.

## 4. Discussion

This study provides compelling evidence that the tested prebiotic formulation P4 (AnXietate) exhibits both neuroprotective and anti-inflammatory properties in a zebrafish model of SCOP-induced cognitive impairment. The behavioral improvements observed in the NTT test, Y-maze, and NOR test were accompanied by a significant reduction in proinflammatory cytokines (IL-6 and IL-1β) and the regulatory cytokine IL-10 in LPS-stimulated PBMCs, suggesting a multifactorial mechanism of action involving both central and peripheral modulation.

The reduction in IL-6 and IL-1β supports a selective immunomodulatory effect, possibly mediated via suppression of upstream signaling pathways such as NF-κB. IL-6 is a key cytokine in systemic inflammation and is associated with anxiety and cognitive impairment in both clinical and experimental models [5,10,19]. IL-1β, on the other hand, is closely linked with neurodegenerative processes and is known to impair synaptic plasticity and neurogenesis, contributing to deficits in learning and memory [5,6].

The concurrent reduction in IL-10, an anti-inflammatory cytokine, may represent a compensatory response to diminished pro-inflammatory signals or reflect a broader shift in immune homeostasis. Together, these findings suggest that the formulations may attenuate inflammation in a targeted manner, potentially improving neural resilience without broadly suppressing immune function.

This interpretation is supported by behavioral data showing that both P1 and P4 significantly alleviated SCOP-induced anxiety-like behavior and cognitive dysfunction in zebrafish. SCOP is widely used to induce cholinergic blockades and oxidative stress, mimicking the cognitive deficits seen in neurodegenerative disorders such as AD [13,14]. It is important to note that the present study employed a SCOP concentration of 100 µM, which differs significantly from the higher doses used in previous work. For example, Hamilton et al. [13] reported that behavioral alterations, particularly reductions in anxiety-like behavior in zebrafish, were not evident until a much higher concentration of 800 µM was administered (see Figure 2 in their study). In contrast, our findings demonstrate that even at 100 µM, SCOP reliably induced anxiety-like responses and memory impairment in zebrafish. These discrepancies may be attributed to differences in experimental design, zebrafish strain, behavioral endpoints, or sensitivity to scopolamine exposure. The use of a lower dose in our study emphasizes the robustness of our model and supports its suitability for detecting subtle neurobehavioral alterations and the therapeutic effects of prebiotic interventions. In interpreting the behavioral effects of SCOP, it is important to consider interspecies differences in neurochemical systems and behavioral repertoires. SCOP’s behavioral effects may differ across species due to variation in cholinergic system architecture and functional organization. For example, in rodents, SCOP reliably induces anxiogenic responses, while in zebrafish, its effects on anxiety-like behavior are more variable and strongly influenced by the testing context and behavioral paradigm employed [13]. These discrepancies highlight the complexity of modeling cholinergic dysfunction across species and underscore the importance of cautious interpretation when extrapolating zebrafish findings to mammalian systems.

The behavioral data presented in this study demonstrate the neuroprotective and anxiolytic potential of prebiotic formulations P1 and P4 in a SCOP impairment. The number of entries in the top zone, the time spent in the top zone, and the latency to first entry are well-established measures of anxiety-like behavior in zebrafish. A higher frequency of top-zone entries and reduced latency are typically interpreted as anxiolytic effects. In the NTT, which is widely used to assess anxiety-related responses in zebrafish, P4 at 6 mg/L resulted in a notable reduction of anxiety-like behavior, as indicated by increased time in the upper region and decreased freezing duration. However, P4 at 3 mg/L did not produce significant anxiolytic effects, highlighting a possible non-linear dose–response relationship, which is frequently observed with natural products and gut–brain axis modulators [5,11]. The discrepancy between doses suggests that a minimal concentration threshold may be required to activate key neuromodulator pathways or gut microbiota interactions that influence zebrafish behavior.

Interestingly, P1 at 3 mg/L did not significantly reduce anxiety-like behavior in the NTT, whereas the 6 mg/L dose exhibited clear anxiolytic effects. This observation suggests a potential non-linear dose–response relationship, where the optimal anxiolytic action of P4 is achieved at a higher concentration. Such non-monotonic responses are common in neurobehavioral studies, particularly with natural compounds and microbiota-targeting interventions. It is also important to note that not all behavioral endpoints responded uniformly to the lower dose of P4, indicating that certain aspects of anxiety and cognition may require different thresholds of neuromodulator activity to elicit measurable effects. These findings emphasize the need for careful dose optimization in the development of prebiotic-based neurotherapeutics.

Moreover, in the Y-maze test, both P1 and P4 at 6 mg/L significantly enhanced spontaneous alternation percentage, indicating their potential to reverse SCOP-induced memory impairments. Importantly, while P4 (6 mg/L) was associated with higher alternation rates, this effect cannot be solely attributed to increased locomotion. Despite showing elevated distance traveled, no significant differences were observed in overall locomotor activity across treatment groups, and tracking data revealed that fish in the P4 (6 mg/L) group showed a strong affinity for the novel arm, suggesting goal-directed exploratory behavior rather than hyperactivity. These findings are consistent with previous research showing that increased alternation in zebrafish can reflect improved cognitive flexibility and recognition memory, provided it is not confounded by general hyperlocomotion [25,26].

The NOR test further supported these conclusions. Both P1 and P4 at 6 mg/L significantly increased preference for the novel object, confirming their positive effect on recognition memory. Interestingly, P3 at 6 mg/L showed a trend toward improved cognitive performance, but the effect did not reach statistical significance, indicating a more limited or variable efficacy for this formulation. These results may be due to differences in composition or bioavailability between formulations.

Taken together, these findings reinforce the role of gut microbiota modulation in behavior regulation, particularly under conditions of cholinergic impairment. SCOP disrupts muscarinic receptor signaling and impairs cholinergic neurotransmission, processes that are closely tied to cognition and mood regulation [13,15].

The ability of prebiotic formulations to counteract these effects suggests involvement of microbiota-mediated pathways, potentially through reduced neuroinflammation, restored neurotransmitter balance, or modulation of brain-derived neurotrophic factors (BDNF), though these mechanisms warrant further investigation.

The neuroprotective effect of prebiotics may result from the suppression of neuroinflammation, as inflammatory cytokines such as IL-1β and IL-6 are known to modulate neural circuits involved in emotional regulation and memory formation [6,10].

The anxiolytic and cognitive-enhancing properties observed in this study are in line with prior findings demonstrating the efficacy of natural and microbiota-modulating compounds in reversing SCOP-induced impairments in zebrafish [16,17]. For example, *Solanum macrocarpon* and *Origanum majorana* extracts were shown to mitigate behavioral alterations via antioxidant and anti-inflammatory mechanisms [16,17]. These parallels further support the involvement of the gut–brain axis in mediating the observed effects.

Additionally, the prebiotic formulation P4 exhibited concentration-dependent effects on HT-29 cell viability. A low concentration (2%) enhanced viability, possibly through stimulation of metabolic activity or cell proliferation, which may benefit gut barrier function and epithelial health. In contrast, a high concentration (10%) resulted in marked cytotoxicity, suggesting cellular stress or toxicity rather than purely anti-inflammatory effects. This biphasic response is typical of bioactive phytocompounds and highlights the importance of dose optimization to balance efficacy and safety [11,19].

Notably, the marked reduction in cytokine levels at 10% P4 may reflect nonspecific suppression of immune responses due to cytotoxicity rather than regulated immune modulation. This raises concerns about the safety of high-dose application, particularly in vulnerable systems or chronic conditions where epithelial or immune function may already be impaired. Conversely, the 2% concentration appears both safe and effective, offering anti-inflammatory benefits without compromising viability, an important consideration for potential long-term therapeutic use.

Collectively, the current findings suggest that the anxiolytic and cognitive benefits observed in zebrafish are, at least in part, mediated through the anti-inflammatory action of the prebiotic formulations. By reducing levels of IL-6 and IL-1β—both central players in neuroimmune signaling—the prebiotics may help restore the neurochemical and structural integrity disrupted by SCOP-induced neurotoxicity [6,10,13]. Given the increasing recognition of the gut–brain axis in regulating mood and cognition [5,6,7], these results reinforce the potential of microbiota-targeting interventions in managing neuropsychiatric disorders.

One limitation of this study is the absence of a baseline control group unexposed to SCOP. As a result, the intrinsic effects of P1 and P4 on anxiety-like behavior and cognitive performance in healthy zebrafish could not be fully assessed. Future investigations should include non-SCOP-treated controls to better delineate the independent behavioral profiles and potential nootropic or anxiolytic properties of these formulations under physiological conditions. Additionally, while the current findings suggest that the tested prebiotic formulations exert anxiolytic and cognitive-enhancing effects in the zebrafish model, caution must be exercised when considering their translational potential to mammalian systems. Given the noted interspecies variability in behavioral and neurochemical responses to SCOP and other pharmacological agents, it is possible that prebiotics may not exhibit the same degree or type of anxiolytic effects in mammals. The neurophysiological and microbiota–gut–brain axis differences between fish and mammals may influence how such formulations interact with neural pathways involved in anxiety and cognition. Therefore, further studies in rodent or higher-order mammalian models are warranted to validate the observed effects and clarify their relevance for human health applications.

Finally, P1 and P4 prebiotic formulations demonstrate a dual role in modulating inflammation and promoting cognitive function, with a concentration-dependent safety profile. Further investigations should examine the specific microbial shifts induced by these formulations and elucidate their downstream neurochemical and metabolic pathways to better understand their therapeutic potential.

## 5. Conclusions

This study demonstrates that the prebiotic formulations P1 (ColonX) and P4 (AnXietate) significantly counteract SCOP-induced anxiety-like behavior and cognitive impairments in adult zebrafish. These behavioral improvements were accompanied by a notable reduction in pro-inflammatory cytokines (IL-6 and IL-1β) and improved cell viability at optimized concentrations, highlighting the formulations’ dual neuroprotective and anti-inflammatory properties. The concentration-dependent effects observed in vitro further emphasize the importance of dosage in clinical applications. Taken together, these findings suggest that microbiota-targeting prebiotic interventions may offer promising therapeutic avenues for managing neuropsychiatric and neuroinflammatory disorders.

## Data Availability

Data supporting reported results are available from the corresponding authors upon reasonable request.
